# Voyages in Vacation.—IV

**Published:** 1888-10-20

**Authors:** 


### Voyages in Vacation.—IV.

By One in Search of Restful Restoratives.

*(Continued from page 29.)*

### WHERE THE PLEASURE YACHTS ARE AT PRESENT DEFICIENT.

In a previous article we mentioned that the proposal to send the *Victoria* on a voyage round the world proved impracticable owing to the comparatively small number who entered for it. The reason is not far to seek. Apart from the expense and time, few people who are thoughtful would place themselves for so long a period, unless specially protected, at the mercy of their fellow-passengers and the owners of a pleasure yacht. Should the companions prove agreeable, the food might become unendurable, and in the latter case there would be no redress, owing to the difficulty of leaving the yacht and returning home. On the shorter voyages a passenger, if uncomfortable or dissatisfied, can leave the vessel at one of the ports of call, but once started on a voyage round the world, all freedom of action in this respect would be voluntarily relinquished by becoming impracticable. On this question of food there is much to be said. The *Victoria*, for instance, had obtained a deservedly good name in contradistinction to other vessels in the same business until the Baltic cruise. Whether the fact that this trip was to be the last of the season affected the dicipline of the men, whether the relative smallness of the number of passengers influenced the owners in provisioning the ship, or whether other causes operated of which we have no knowledge, the fact remains that the food sent to table was so served as to be uneatable on several occasions, even by hungry and strong men, whilst the less robust and squeamish suffered in some cases for want of food. More than one lady had to make meal after meal on biscuits and scraps, and more than once became faint and ill for want of proper food. With the more extravagant passengers the remedy was to take as many meals as possible on shore when in port, with the less opulent discontent and discomfort were the fruits of this mistaken and short-sighted policy in regard to the table.

To supply a few passengers with wholesome food in appetising fashion is so simple a matter that there is no excuse for any owner who fails to fulfil this part of his contract. Paying, as passengers do, from £2 to £2 10*s.* per head per diem, it is inexcusable (1) to supply eggs which have to be sent away in succession because they are offensive to eye and sense as well as uneatable, (2) to send fish to the table which is unpalatable and sometimes distinctly bad and offensive, (3) to serve cold meat in a manner which renders it impossible to touch it, (4) to make coffee and tea quite nauseous in taste, (5) to give salt butter in lieu of fresh where passengers are foolish or careless enough to pass it without remonstrance, and (6) to place on the table at dinner-time an elaborate menu which when tested proves very dust and ashes. Such a policy is as short-sighted as it is indefensible, for a pleasure yacht must depend upon its good management in these respects for its popularity, and without popularity financial success can never be secured by passenger ships of any class. Nothing could have been better than the ship *Victoria*, or the discipline and general arrangements. Yet the cooking and cuisine on the Baltic voyage disgusted even her friends, and necessarily made the ordinary passenger home-sick and uncomfortable, seeing that the food and cooking are really serious considerations, especially when one's sailing powers are, to say the least, not good. Nothing but want of administrative capacity would permit as good and comfortable a yacht as could be wished for by anyone to so belie its record and injure its reputation. Yet this was done in the case of the *Victoria* on the Baltic cruise owing to non-attention to a matter of detail. For it is a matter of detail to provide well-cooked and wholesome food on board ship. Some of the best dinners we have ever eaten have been served on an Atlantic steamer, even in bad weather.

Having every wish to encourage voyages in vacation, we advise those who wish to enjoy such a trip to look especially to three cardinal points: good food, agreeable companions, and fine weather. With these three essentials present a voyage leaves nothing to be desired. We have taken some pains to discover the cause of bad cooking and defective food supply on board ship. It seems to be certain that if an owner adopts the policy of purchasing all his stores at the port of departure, instead of relying upon the fresh fruits, vegetables, and other supplies he will be able to procure at the ports of call, he will fail to give satisfaction all round. An ice-chamber is all very well, but it cannot keep everything decently fresh and palatable throughout a lengthened cruise. Besides, many things are better obtainable at ports of call than they are at home. Again, it is a mistaken policy to attempt too much. Passengers don't want a grand array of so-called French dishes; all they care for is good and wholesome food, well cooked and temptingly sent to table. This can easily be secured if foresight is exercised by the management. Here is a little practical point for guidance. Passengers will be well advised to ascertain how joints are sent to table. Does the boat contain a table in the saloon, with steam-heated dishes from which the joints can be carved and served? If not, our advice is, don't sail in her.

To have fowls fish, joints, &c., cut up in the galley, placed in an oven afterwards, and then sent to table as wanted, is to secure dissatisfaction and discomfort in advance. Food so served must be sodden, unpalatable, present an untempting appearance, and cause an immense waste. Even the most elementary knowledge of cooking will confirm this view, and its truth was forcibly brought to the notice of the passengers of the *Victoria*. Until French cooks are given up in favour of capable Englishmen, and a carving-table of an approved pattern is placed in the saloon, it will be impossible to give satisfaction, because the meals will never be well served. It is a short-sighted policy and proves an absence of administrative capacity to instruct or allow the purser of one of these yachts to purchase second-class articles. It is better, for example, to give no fruit than to give apples which when served have to be sent away uneaten, or plums that are only fit for cooking. The same may be said of a system which provides one stewardess to attend to all the ladies out of seventy passengers, or which permits the same stewardess to act as nursemaid to the captain's and owner's children, and as maid to the wives of the same persons when they are aboard. A sea voyage under the most favourable circumstances must result in sickness and suffering at times to several of the passengers, if not to the majority. Hence this possibility should be recognized when the vessel is commissioned, so that the cooking and attendance shall be adequate to meet it properly, and the abundant supply of all sorts of comforts and tempting foods when passengers are ill a matter of ordinary routine. To provide the few passengers who are not ill with food they cannot eat, or to supply those who are ill with messes they cannot touch, is little short of disgraceful. It ought to be unnecessary to state that the tea and coffee served should be both hot and palatable. A concoction of mouse-flavoured lukewarm water, even when coloured to resemble one or other of these beverages, if served in the saloon, must excite the wrath of the meekest passenger. All administrative neglect and absence of proper supervision must result in financial loss. Any owners who permit such evils in their boats are mere amateur caterers who deserve to lose their money. This reflection is but a small consolation to passengers who have experienced the discomfort and annoyance of paying fifty shillings a-day for something which was worth only about half of that sum. No doubt the continuance of such losses, by making owners realise this wholesome fact, may ultimately produce a pleasure yacht which shall be admirably administered and an unmixed pleasure to travel in. Till this happens tourists will be well advised to stick to the tours arranged by the leading lines of ocean steamers, especially if they contemplate a voyage round the world.

*(To be continued.)*

